# Brain border‐derived CXCL2 ^+^ neutrophils drive NET formation and impair vascular reperfusion following ischemic stroke

**DOI:** 10.1111/cns.14916

**Published:** 2024-08-12

**Authors:** Tingting Huang, Yunlu Guo, Wanqing Xie, Jiemin Yin, Yueman Zhang, Weijie Chen, Dan Huang, Peiying Li

**Affiliations:** ^1^ Department of Anesthesiology, Clinical Research Center, Renji Hospital Shanghai Jiao Tong University School of Medicine Shanghai China; ^2^ Key Laboratory of Anesthesiology, Ministry of Education Shanghai Jiao Tong University Shanghai China

**Keywords:** brain border, neutrophil, reperfusion, stroke

## Abstract

**Background:**

The brain border compartments harbor a diverse population of immune cells and serve as invasion sites for leukocyte influx into the brain following CNS injury. However, how brain‐border myeloid cells affect stroke pathology remains poorly characterized.

**Methods and Results:**

Here, we showed that ischemic stroke‐induced expansion of CXCL2^+^ neutrophils, which exhibit highly proinflammatory features. We tracked CXCL2^+^ neutrophils in vivo by utilizing a photoconvertible Kik‐GR mouse (fluorescent proteins Kikume Green Red, Kik‐GR) and found that brain‐infiltrating CXCL2^+^ neutrophils following ischemic stroke were mainly derived from the brain border rather than the periphery. We demonstrated that CXCL2 neutralization inhibited the formation and releasing of neutrophil extracellular traps (NETs) from in vitro cultured primary neutrophils. Furthermore, CXCL2‐neutralizing antibody treatment reduced brain infarcts and improved vascular reperfusion at day 3 postischemic stroke.

**Conclusions:**

Collectively, brain border‐derived CXCL2^+^ neutrophil expansion may impair vascular reperfusion by releasing NETs following ischemic stroke.

## Graphical abstract

CXCL2^+^neutrophils at the border of the brain are rapidly generated and infiltrated into the brain following cerebral ischemia to promote the production of neutrophil extracellular traps, exacerbating the no‐reflow phenomenon and hampering recovery after cerebral ischemia.

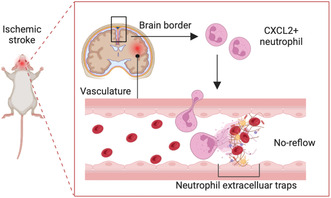

## 1 INTRODUCTION

Immune cells capable of interacting with brain‐resident cells at the border of the central nervous system (CNS) play a key role in shaping neuroimmune responses and leading to neurodegenerative diseases.^1^, ^2^ Recently, accumulating evidence suggests that niches bordering the brain (including the skull, meninges) are the parenchymal myeloid cell reservoirs of the CNS.^3^ Following brain injury and neuroinflammation, channels between the skull bone marrow and meninges allow myeloid cells to enter the CNS parenchyma, and these myeloid cells display a different phenotype than blood‐derived myeloid cells.^4^

Stroke is a leading cause of death and disability worldwide.^5^ Previous studies have reported that up to 25% of ischemic stroke patients do not fully regain function despite treatment with thrombolysis or mechanical thrombectomy. This phenomenon is often referred to as reperfusion failure or no reflow.^6^ Multiple evidence suggests that the pathological cause of no‐reflow is the adhesion, aggregation, and rolling of blood components along the endothelium and capillary blockage by neutrophils.^7^, ^8^ However, trials targeting neutrophils have shown modest benefits in stroke treatment.^9^, ^10^, ^11^

Recent studies have demonstrated that the skull has great potential as a site for diagnosing, monitoring, and treating brain diseases.^4^ In the stroke state, the skull displays a unique transcriptomic profile compared with other bones, with mice exhibiting late‐stage neutrophil phenotypes. However, whether brain border neutrophils infiltrate brain tissue and how brain border neutrophils participate in the acute phase of ischemic stroke remain unclear.

Here, we compared single‐cell RNA sequencing data of skull, meningeal, and femur and found that stroke triggered the generation of CXCL2^+^ neutrophils from meningeal. Using the photoconvertible Kik‐GR transgenic mouse model, we showed that CXCL2^+^ neutrophils were mainly derived from meningeal but not peripheral blood. We reported a key role of CXCL2^+^ neutrophils in the formation of neutrophil extracellular traps (NETs) in the acute phase after reperfusion injury of stroke, resulting in worsened neurological outcomes. Treatment with CXCL2 inhibitor may improve cerebrovascular reperfusion and reduce ischemic brain injury.

## 2 METHOD

### 2.1 Animals

Male C57/BL6 mice (8–10 weeks old) were purchased from Shanghai SLAC Laboratory Animals and bred in accordance with conventional laboratory settings (22°C, a 12‐h light–dark cycle, and free access to food and water). For Kik‐GR transgenic mouse, we sincerely appreciate for the gift of Professor Duan Shengzhong from Shanghai Ninth People's Hospital. All experiments were approved by the Renji Hospital Institutional Animal Care and Use Committee and performed in accordance with the Institutional Guide for the Care and Use of Laboratory Animals.

### 2.2 Ischemic stroke model

Induction of transient middle cerebral artery occlusion (tMCAO) was performed according to previously described details^12^ and previous experiments from our laboratory.^13^ Mice were anesthetized with 2% isoflurane in a 30%O_2_/70%N_2_O gas mixture. After a midline cervical incision, a monofilament was inserted into the left common carotid artery and advanced to occlude the origin of the middle cerebral artery. After 60 min, the occlusive wire was removed to allow for reperfusion. Sham‐operated animals received the same anesthesia and surgical incision but did not occlude the middle cerebral artery. During the procedure, body temperature was maintained at 37 ± 0.5°C with a heating pad.

### 2.3 CBF measurement

Cerebral blood flow (CBF) was measured using the PeriCam PSI system (Perimed) to confirm vessel occlusion and reperfusion. Speckle contrast is defined as the ratio of the pixel intensity scale to the average pixel intensity. Spot visibility relative to light‐scattering particle velocity is converted to a correlation time value that is inversely proportional and linear to the mean blood flow velocity. CBF changes were expressed as a percentage of pre‐tMCAO baseline. Mice with at least a 70% reduction in regional CBF during occlusion compared with preischemic baseline levels were excluded from further experiments.

### 2.4 Behavioral tests

Sensorimotor function was assessed using the modified Garcia score, which was assessed before and after surgery by researchers who kept the confidentiality of experimental group allocation, as previously described.^14^ The Modified Garcia Score is a well‐established sensorimotor assessment system consisting of five separate tests, one measuring sensory function and four measuring motor function. Each test is scored on a scale of 0 to 3 points (maximum score = 15 points): (a) body proprioception, (b) forelimb walking, (c) limb symmetry, (d) lateral turning, and (e) as above said climb.

### 2.5 Immunofluorescence

Euthanize mice by perfusion with ice‐cold saline, and then fix the brain tissue with 4% paraformaldehyde (PFA) through the left ventricle. The extracted brain tissue was fixed in 4% PFA for 24 h and dehydrated in 30% sucrose solution for 48 h/twice at 4°C. The brain tissue was then embedded in OCT (Sakura) and cryosectioned at a thickness of 25 μm. Brain sections were incubated with 10% normal donkey serum in PBS containing 0.1% Triton X‐100 for 30 min at room temperature, followed by incubation with appropriate primary antibodies in the same buffer overnight at 4°C. The anti‐Iba‐1 (1:500, catalog: ab178846, Abcam), anti‐NeuN (1:500, catalog: MAB377, Merck), anti‐GFAP (1:500, catalog: ab68428, Abcam), anti‐Ly6g (1:500, catalog:127602, Biolegend), anti‐CXCL2 (1:500, catalog:MAB452, R&D system), anti‐H3cit (1:500, catalog: ab5103, Abcam), anti‐MAP2 (1:500, catalog: ab32454, Abcam), and anti‐CD31 (1:500, catalog: 3628, R&D system) primary antibodies were used. After primary antibody incubation, brain sections were washed three times at room temperature, followed by incubation with appropriate fluorescent‐labeled secondary antibodies (1:8000) for 1.5 h at room temperature. All the confocal images were captured on a laser scanning confocal microscope (Olympus Fluoview FV3000, Olympus). The numbers of target‐positive cells were quantified by a blinded investigator using NIH Image J (1.52a). Three randomly selected microscopic fields in the cortex on each section were analyzed for each brain by a blinded investigator. The immune‐positive cells were presented as the mean percentage of cells per field. Since neutrophils are predominantly located in the core of the infarct, the principle of the neutrophil counting region was in the infarct core area for statistics.

### 2.6 Flow cytometry

For brain tissues, we homogenized the hemisphere ipsilateral to the infarct of tMCAO or sham mice using Neural Tissue Dissociation Kit (130‐093‐231, Miltenyi Biotec) by the gentle MACS Dissociator following the manufacturer's instructions. The immune‐cell‐enriched population was collected using Percoll gradient centrifugation. Isolated cells were stained with anti‐CD45‐PE‐cy7 (catalog: a552848, Biolegend), anti‐Ly6g‐BV421 (127628, Biolegend), anti‐CXCL2 (catalog: BAF452, R&D system).

For single‐cell isolation from calvaria, after the brain was removed, calvaria was cut into small pieces and filtered through 70 μm cell strainers, and then centrifuged at 4°C, with 1000 rpm, for 5 min. The supernatant of all samples was then discarded and the remaining precipitate was resuspended in 50 mL FACS buffer with 0.5 mL FC blocker. The samples were incubated for 20 min, in dark at 4°C. Then 50 mL of antibody mix was added to each sample: anti‐CD45‐PE‐cy7 (catalog: a552848, Biolegend), anti‐CD11b‐APC (catalog: 101212, Biolegend), anti‐Ly6g‐BV421 (127628, Biolegend), and anti‐CXCL2 (catalog: BAF452, R&D system).

Single‐cell suspension was incubated for 30 min on ice in dark. After adding 3 mL of FACS buffer to each sample, the samples were centrifuged at 300×*g* for 5 min at 4°C. After discarding the supernatant, samples were resuspended in 200 mL of FACS buffer to be measured by the BD FACSVerse (BD Bioscience).

### 2.7 Primary neutrophils culture

Primary neutrophils were obtained from the bone marrow of wild‐type mice (8–10 weeks old). The bone marrow of the tibia and femur was rinsed with PBS/1% BSA, filtered through a 40 μm cell strainer, and centrifuged at 300×*g* for 5 min. After removing the supernatant, cells were harvested and isolated using a neutrophil enrichment kit (Stemcell) following the manufacturer's instructions. The isolated neutrophils were cultured under standard culture conditions (relative humidity 95%, 5% CO_2_, 37°C) with the addition of 10% FBS and antibiotics (penicillin 100 UmL^−1^, streptomycin 100 UmL^−1^ (pen/strep), HVD Life Sciences, Inc.).

### 2.8 NETs quantification by ELISA

To quantify NETs, MPO‐DNA complexes were quantified using an in‐house ELISA method according to details previously described.^15^ After coating with anti‐MPO antibody (2 μg/mL; Ab9535, Abcam) overnight at 4°C, 96‐well plates were blocked with 2.5% BSA in PBS for 2 h at room temperature. After three washes, 50 μL of sample and 80 μL of incubation buffer containing peroxidase‐labeled anti‐DNA mAb (Cell Death ELISAPLUS, 11774425001, Roche) were added to the wells. Incubate the plate for 2 h at room temperature with shaking at 300 rpm. After three washes, 100 μL of peroxidase substrate (ABTS) was added. After incubation in the dark at room temperature for 30 min, the absorbance was measured at a wavelength of 405 nm. Values for soluble NET formation were expressed as the percentage increase in absorbance compared to the control.

### 2.9 Photoconversion

Kik‐GR transgenic mice were anesthetized with isoflurane plus oxygen (1%–3% vol, inhalation). Photoelectric conversion was performed using a violet laser light source (405 nm, MTO, China). Place the mouse in the prone position and make a 2‐cm incision on the trunk. Irradiate the exposed vertex for 5 min and then suture the skin.

### 2.10 Single‐cell RNA sequencing (scRNA‐seq) analysis

10x Genomics scRNA‐seq data were obtained through the Gene Expression Omnibus (GEO) database (https://www.ncbi.nlm.nih.gov/geo/, accession number is GSE192616). We used preprocessed 10× backup matrices (barcodes, genes, and matrices) of calf, femur, and meningeal samples under sham and MCAO (3 days) conditions. Seurat objects were created using Seurat version 4.3.0 for further analysis in R version 4.3.1. Cells with a cell count of more than 500 and mitochondrial gene expression of less than 20% (calf and femur) or 30% (meninges) passed the cell quality screen. The integrated dataset was log‐normalized and scaled. A principal component analysis (PCA) was constructed based on the proportion data of all highly variable genes. The first 30 principal components were used for *t*‐distributed stochastic neighbor embedding (tSNE) construction and unsupervised cell clustering (resolution = 0.2).

To identify marker genes, we used the FindAllMarkers (multi‐group comparison) and FindMarkers (two‐group comparison) functions and performed the Wilcoxon Rank‐Sum test with the following test criteria: LogFC > 0.25; *p* < 0.05; min.pct >0.25. Pathway enrichment analysis was performed based on gene ontology (GO) using clusterProfiler version 3.16.1 to identify important pathways of differential genes.

To screen the expression profile of infiltrating neutrophils in the ischemic brain, we obtained scRNA‐seq data from the GSE database (GSE210986‐MCAO3d, GSE174574‐MCAO1d). We used preprocessed barcodes and matrices of brain samples under sham and MCAO conditions. We created a Seurat object using Seurat version 4.3.0 for further analysis in R version 4.3.1.

The integrated dataset was log‐normalized and scaled. A principal component analysis (PCA) was constructed based on the proportion data of all highly variable genes. The first 20 principal components were used for *t*‐distributed stochastic neighbor embedding (tSNE) construction and unsupervised cell clustering (resolution = 0.2).

The neutrophil maturation signatures (Retnlg, Ccl6, S100a6, Clec4d, Prr13, Cebpb, Slpi, S100a11, Btg1, Cxcr2, Fth1, Grina, Mmp8, Fxyd5, Msrb1, H2‐D1, Anxa2, Mmp9, Ftl1, Map1lc3b, Tmcc1, Sat1, Cyp4f18, Junb, Mxd1, Stk17b, Ypel3, Selplg, Il1f9, Dusp1, Slc16a3, Ccr1, Rdh12, Clec4e, Arg2, Cd300ld, Amica1, Ctsd, Gda, Hacd4, Timp2, Fpr1, Ifi27l2a, Slc7a11, Stfa2l1, Il1b, Asprv1, Cxcl2, Gm5483, and Ifitm1) were derived from ref.^16^ The proinflammatory signatures of neutrophil (Il6, Il1a, Il1b, Ifng, Il11, Il7d, Il7f, Il18, and Tnf) were adopted from previous literature.^4^

### 2.11 Statistical analysis

All statistics were performed using statistical test methods in GraphPad Prism v9 or the corresponding R package. First, a Shapiro–Wilk normality test was performed on all datasets. Pairwise comparisons between two groups were performed using two‐tailed Student's *t*‐test. Data from three or more groups were analyzed using one‐way analysis of variance, and Bonferroni posttest was used for multiple comparisons. Multiple comparison procedures used post hoc Tukey's tests to identify specific between‐group differences. Results are expressed as mean ± SD.

## 3 RESULTS

### 3.1 Ischemic stroke‐induced expansion of brain border‐derived neutrophils

**FIGURE 1:**
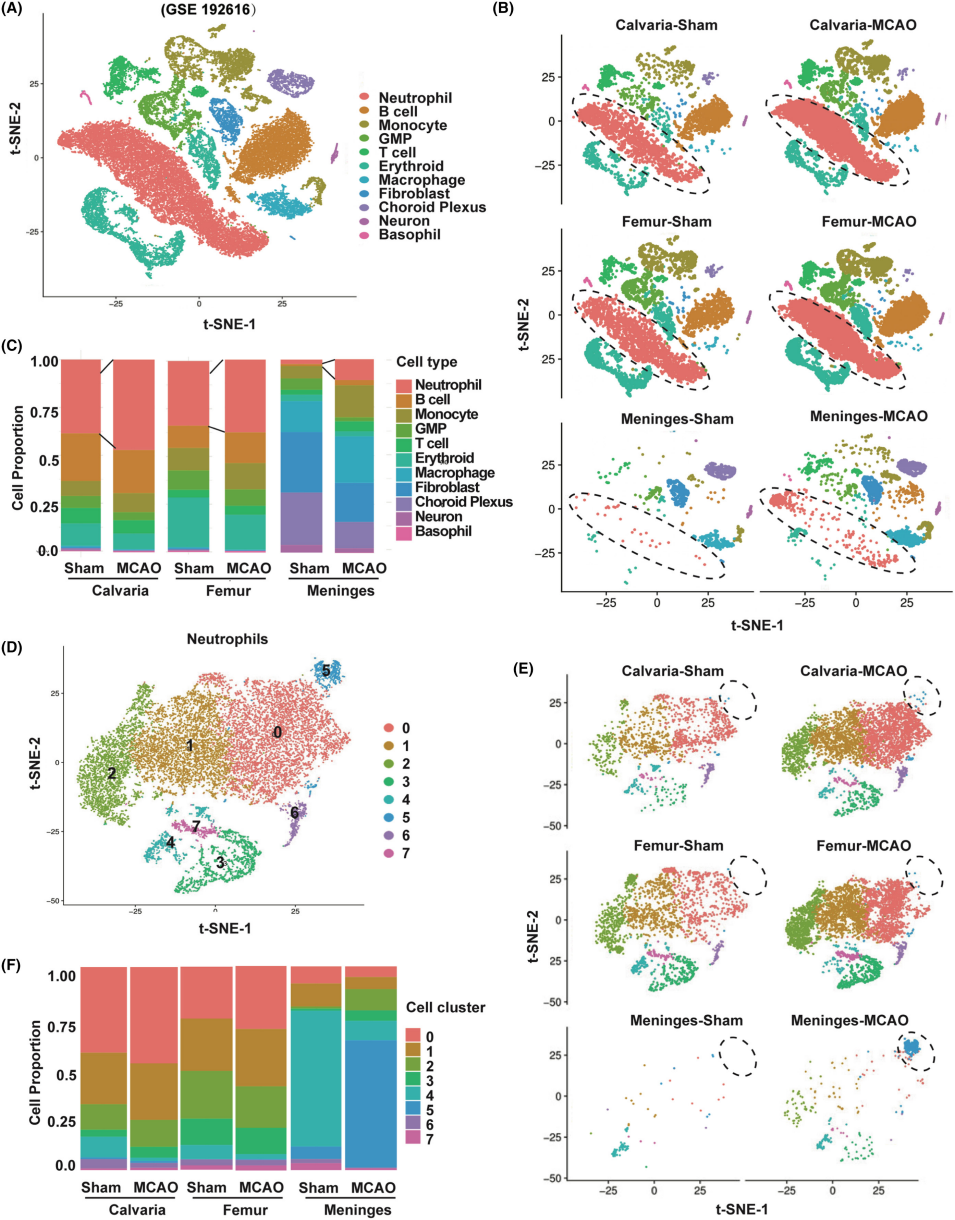
Ischemic stroke‐induced neutrophils expansion in the brain border (A) T‐distributed Stochastic Neighbor Embedding (t‐SNE) distribution of scRNA‐seq, colored by cell type with fine annotated cell types (GSE192616); (B) t‐SNE distribution of scRNA‐seq colored by cell type with fine annotated cell types in sham and MCAO condition; (C) relative proportions of cell types from calvaria, femur, and meninges in sham and MCAO condition; (D) t‐SNE showing the distribution of each subtype of neutrophils; (E) t‐SNE distribution of scRNA‐seq colored by subtype of neutrophils in sham and MCAO condition; (F) relative proportions of each subtype of neutrophils from calvaria, femur, and meninges in sham and MCAO condition.

To investigate the difference in the response to ischemic brain injury of CNS border and peripheral bone marrow (BM), we employed single‐cell RNA sequencing data from GEO database (GSE192616).^4^ Middle cerebral artery occlusion model (MCAO) was established for experimental ischemic stroke. Single‐cell isolation from the femur, calvaria, and meninges was performed in one animal at a time. Six MCAO‐operated and three sham‐operated animals were pooled in threes for single‐cell RNA sequencing (Figure 1A,B). We identified clusters including neutrophil, monocyte, B cell, T cell (cluster 5), GMP, erythroid progenitor, erythroid, macrophage, fibroblast, choroid plexus, basophil, and neuron based on signature genes expressed in each cluster (Figure 1A). Hierarchical clustering showed that femur and calvaria clustered together while meninges separated (Figure 1B). To assess changes in cell populations after MCAO, we compared the percentage of each cell type in the mouse brain 3 days after sham or MCAO procedure. We found that neutrophil was the most changed population in terms of cell proportion following MCAO (Figure 1C).

To investigate whether stroke‐induced neutrophil mobilization differs between peripheral bone marrow and the CNS border, we focused our analysis on neutrophils data and delineated transcriptionally distinct cell clusters (Figure 1D). Hierarchical clustering showed that neutrophils from femur and calvaria clustered together while those from meninges separated (Figure 1E). By comparing the percentage of each cluster in sham and MCAO conditions, we found that MCAO triggered the generation of cluster 5, which was mainly derived from meninges but not femur or calvaria (Figure 1E,F).

### 3.2 Brain border‐derived CXCL2 ^+^ neutrophils induced by ischemic stroke displayed proinflammatory property

**FIGURE 2:**
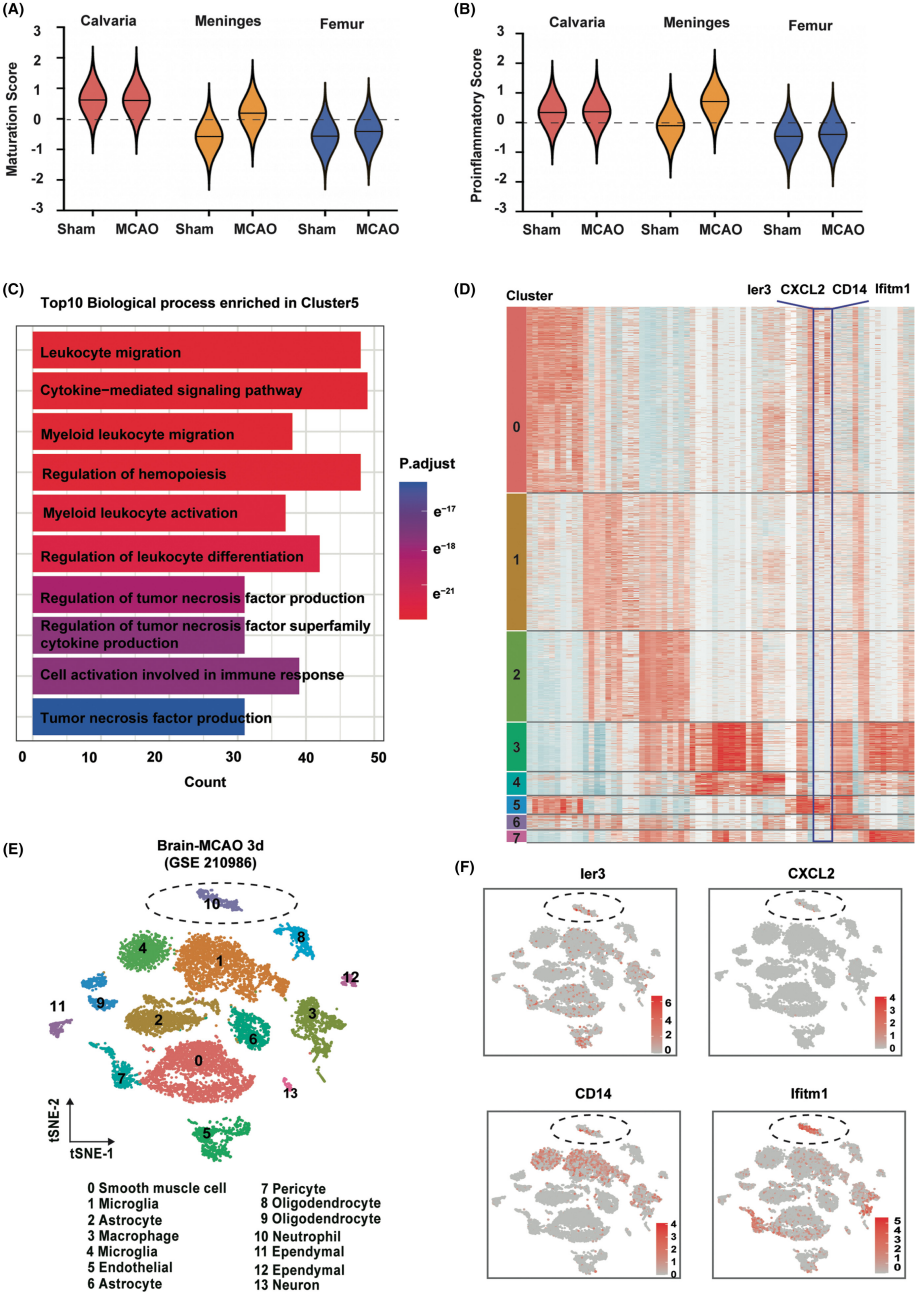
Meninges‐derived CXCL2^+^ neutrophils display proinflammatory property (A) Violin plot of maturation scores for neutrophil subcluster in sham, and MCAO; Maturation score is based on the expression of Retnlg, Ccl6, S100a6, Clec4d, Prr13, Cebpb, Slpi, S100a11, Btg1, Cxcr2, Fth1, Grina, Mmp8, Fxyd5, Msrb1, H2‐D1, Anxa2, Mmp9, Ftl1, Map1lc3b, Tmcc1, Sat1, Cyp4f18, Junb, Mxd1, Stk17b, Ypel3, Selplg, Il1f9, Dusp1, Slc16a3, Ccr1, Rdh12, Clec4e, Arg2, Cd300ld, Amica1, Ctsd, Gda, Hacd4, Timp2, Fpr1, Ifi27l2a, Slc7a11, Stfa2l1, Il1b, Asprv1, Cxcl2, Gm5483, and Ifitm1; (B) mean and standard deviation of proinflammatory score over neutrophils in sham, and MCAO; Inflammatory score is based on the expression of IL6, IL1α, IL1β, IFN‐γ, IL11, IL7d, IL7f, IL18, and TNF; (C) gene ontology analysis of DEGs for cluster5; (D) heatmap showing the row‐scaled expression of the 10 highest DEGs per cluster for neutrophils; (E) UMAP plot showing scRNA‐seq transcriptomes of brain cells (total 11,226 cells) from sham and MCAO 3d mice (GSE210986); (F) expression of cluster 5 signature gene (Ier3, CXCL2, CD14, and Ifitm6) in clusters.

To characterize meninges‐derived neutrophils, we next examined the maturity and proinflammatory property of neutrophils from calvaria, meninges, and femur. We found that neutrophils from calvaria displayed late‐stage profiles while neutrophils from femur displayed early‐stage profiles (Figure 2A). Notably, the neutrophil maturation process in the calvaria and femur was not significantly altered, while it was markedly changed in the meninges. We found that the neutrophils in the meninges were differentiated into more mature state 3 days following MCAO (Figure 2A). Consistently, neutrophils from calvaria showed higher proinflammatory score than that from femur (Figure 2B), while the proinflammatory property of neutrophils in the calvaria and femur remained unchanged after MCAO. However, the proinflammatory capacity of neutrophils in the meninges was markedly enhanced 3 days following MCAO (Figure 2B).

We next explored the identity of meninges‐derived cluster5 which newly generated after MCAO. Gene ontology (GO) analysis demonstrated that cluster5 showed enrichment in leukocyte migration, cytokine‐mediated signaling pathway, and regulation of tumor necrosis factor production (Figure 2C). To distinguish meninges‐derived neutrophils from peripheral‐derived or skull, different expressed genes (DEGs) analysis demonstrated that Ier3, CXCL2, CD14, and Ifitm1 were the top4 highly expressed genes in the cluster5 compared to other neutrophil clusters (Figure 2D). After screening the expression profiles of these genes in the ischemic brain from database (GSE210986) (Figure 2E), we demonstrated that CXCL2 was specifically expressed in the neutrophil cluster 3 days after MCAO (Figure 2F) while Ier3, CD14, and Ifitm1 also expressed in microglia, macrophage, and pericyte. We thus identified CXCL2 as a potential candidate gene for brain border‐derived neutrophils because of its specific expression profile in brain‐infiltrated neutrophils.

### 3.3 Accumulation of CXCL2 ^+^ neutrophils in the ischemic brain was mainly derived from brain border

**FIGURE 3:**
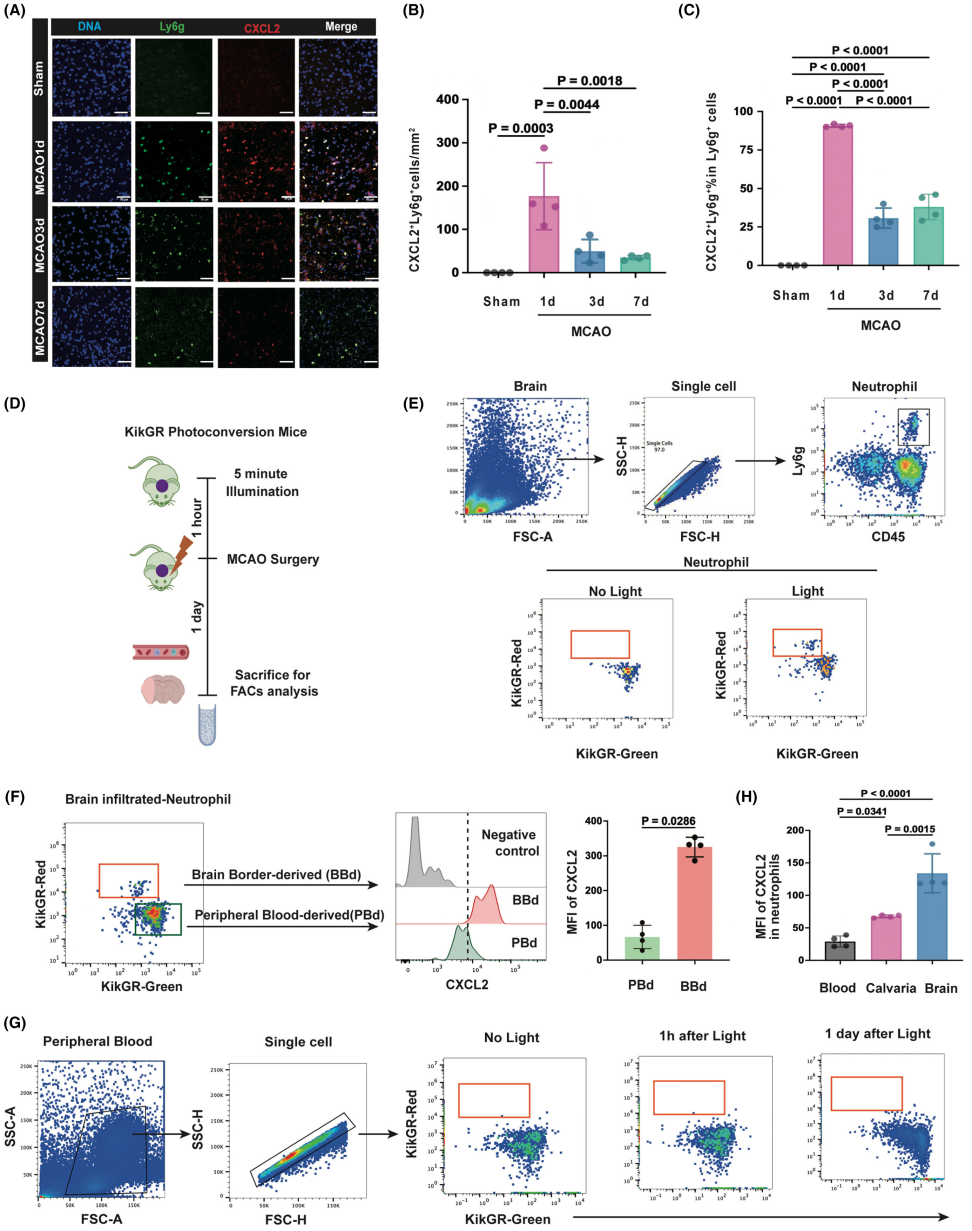
Accumulation of CXCL2^+^ neutrophils in the ischemic brain is mainly derived from brain border (A) representative images of coronal brain slices collected after tMCAO showing the DNA (DAPI, blue) localization of CXCL2 (red) in Ly6g^+^ (green) neutrophils at different time points after tMCAO. Scale bar: 50 μm; (B) quantification of the number of CXCL2^+^Ly6g^+^ cells in the ischemic regions indicated in A, *n* = 4 mice per group; (C) quantification of the percentage of CXCL2^+^Ly6g^+^ cells among infiltrated neutrophils in the ischemic regions indicated in A, *n* = 4 mice per group; (D) photoconversion in Kik‐GR mouse model to track cell trafficking from skull to brain 1 day after MCAO; (E, F) flow analysis of photoconverted neutrophils in the ischemic brain; (G) flow analysis of photoconverted neutrophils in the peripheral blood.

We further validated CXCL2 expression in the single‐cell RNA sequencing results of brain at MCAO1d^17^ (GSE 174574) and found that approximately 90% of neutrophils expressed CXCL2 (Figure S1A,B). To examine the temporal dynamics of CXCL2^+^ neutrophils following MCAO. Immunostaining revealed that CXCL2 was co‐localized with Ly6G‐positive neutrophils in the core infarct area (Figure 3A and Figure S2). The percentage and absolute number of CXCL2^+^ neutrophils increased robustly 1 day after MCAO and began to decrease 3 days after MCAO (Figure 3B,C). In order to investigate the origin of infiltrated CXCL2^+^ neutrophils, we treated Kik‐GR (Photoconvertible fluorescent proteins Kikume Green Red (Kik‐GR) mice which can be irreversibly converted from green to red upon exposure to violet light)^18^ with 5‐minute illumination and then subjected them to MCAO surgery. Peripheral blood and injured brain tissue at 1 day after MCAO were obtained for FACs analysis (Figure 3D). With 405 nm of illumination, brain border‐derived (BBd) neutrophils displayed red fluorescence while peripheral blood‐derived (PBd) neutrophils displayed red fluorescence (Figure 3E). Importantly, BBd neutrophils expressed much higher expression of CXCL2 than PBd neutrophils, suggesting that the accumulation of CXCL2^+^ neutrophils in the ischemic brain was mainly derived from the brain border rather than peripheral blood or BM (Figure 3F). Besides, to verify whether local exposure of the skull would affect circulating neutrophils, we tested the fluorescence signal in the peripheral blood after illumination and found that the skull illumination did not influence circulating neutrophils (Figure 3G). Moreover, CXCL2 expression in the neutrophils from blood, brain border, and brain at 1 day after MCAO was also analyzed. We found that MCAO did not alter CXCL2 expression in the neutrophils from peripheral blood (Figure S3A), but increased that in neutrophils from calvaria (Figure S3B). The level of CXCL2 in the infiltrated neutrophils from brain tissue was the highest followed by calvaria, while neutrophils from blood expressed low level of CXCL2 (Figure 3H).

### 3.4 CXCL2 ^+^ neutrophils were NETosed to form NETs following stroke

**FIGURE 4:**
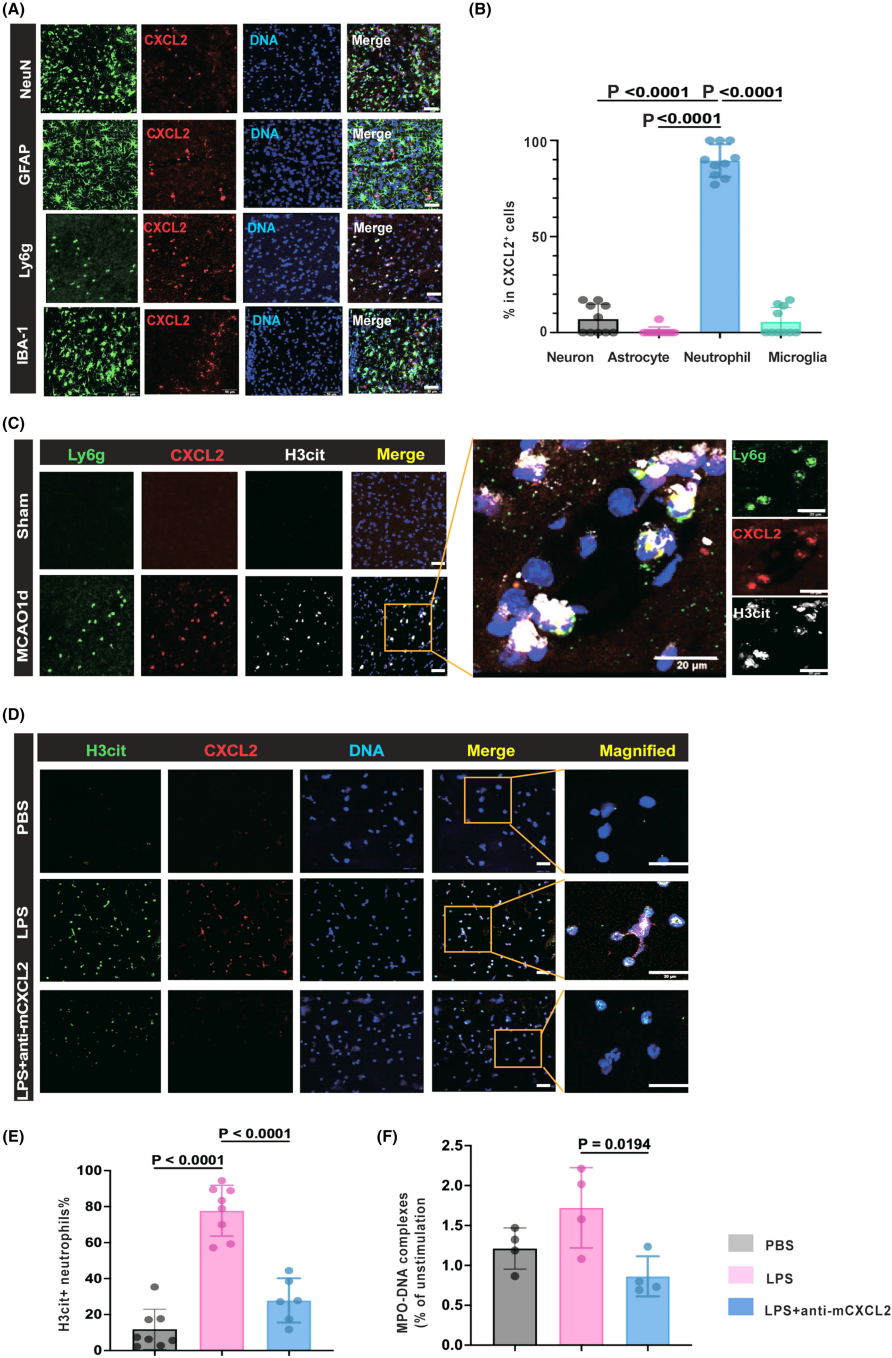
CXCL2^+^ neutrophils are NETosed neutrophils to form NETs (A) representative images of coronal brain slices collected at day 1 after MCAO showing the localization of CXCL2 (red) in neurons (NeuN+), astrocytes (GFAP+), microglia cells (Iba‐1+) in the peri‐infarct and neutrophils (Ly6g+) in the core area. Scale bar: 50 μm; (B) quantification of the CXCL2+ in neurons (NeuN+), astrocytes (GFAP+), neutrophils (Ly6g+), and microglia (Iba‐1+) indicated in A, *n* = 4 mice per group; (C) representative images of H3Cit (white), CXCL2 (red), and Ly6G (green) triple‐positive cells from ischemic mice in the core area at day 1 after MCAO. DNA was visualized with DAPI (blue). Bar = 20 μm; (D) representative images of primary neutrophils isolated from bone marrow and collected after 4 h of culture treated with either CXCL2 neutralization antibody, control antibody showing the DNA (DAPI, blue) localization of CXCL2 (red) and H3Cit (green). Scale bar: 20 μm; (E) quantification of the percentage of H3cit^+^ neutrophils in D; (F) culture medium was collected after 4 h of culture treated with either CXCL2 neutralization antibody or control antibody. MPO‐DNA complexes were measured by ELISA.

Since CXCL2 is involved in the development of cardiovascular diseases through its role as an important inflammatory chemokine,^19^ we next performed immunostaining of CXCL2 in the ischemia brain at 1 day after MCAO and found that infiltrating neutrophils were the predominant producers of CXCL2 followed by microglia, and neurons (Figure 4A,B). Importantly, 89.5% of the CXCL2‐positive cells were Ly6g‐positive neutrophils while CXCL2 was not detectable in astrocytes (Figure 4A,B).

As accumulation of NET exacerbates neuroinflammation and worsen the prognosis of stroke patients,^20^ we sought to investigate whether CXCL2 drives NETs formation by neutrophils. Immunostaining revealed CXCL2 was colocalized with Ly6g‐positive neutrophils and H3cit, a marker for neutrophils undergoing NET formation (Figure 4C). To further identify whether CXCL2 was involved in the formation of NETs, we treated primary neutrophils with CXCL2‐neutralizing antibody (MAB452‐500, 1 μg/mL) after 4 h of LPS stimulation (200 ng/mL). We found that CXCL2 neutralization led to a decreased number of H3cit‐positive neutrophils (Figure 4D,E). Consistently, we detected a significant downregulated level of MPO‐DNA complex in the culture medium of neutrophils treated with CXCL2‐neutralizing antibody in comparison with IgG control treatment (MAB0061‐500, 1 μg/mL) (Figure 4F), suggesting a key role of CXCL2 in mediating NETs formation.

### 3.5 Inhibition of CXCL2 attenuated ischemic brain injury and improved vascular reperfusion

**FIGURE 5:**
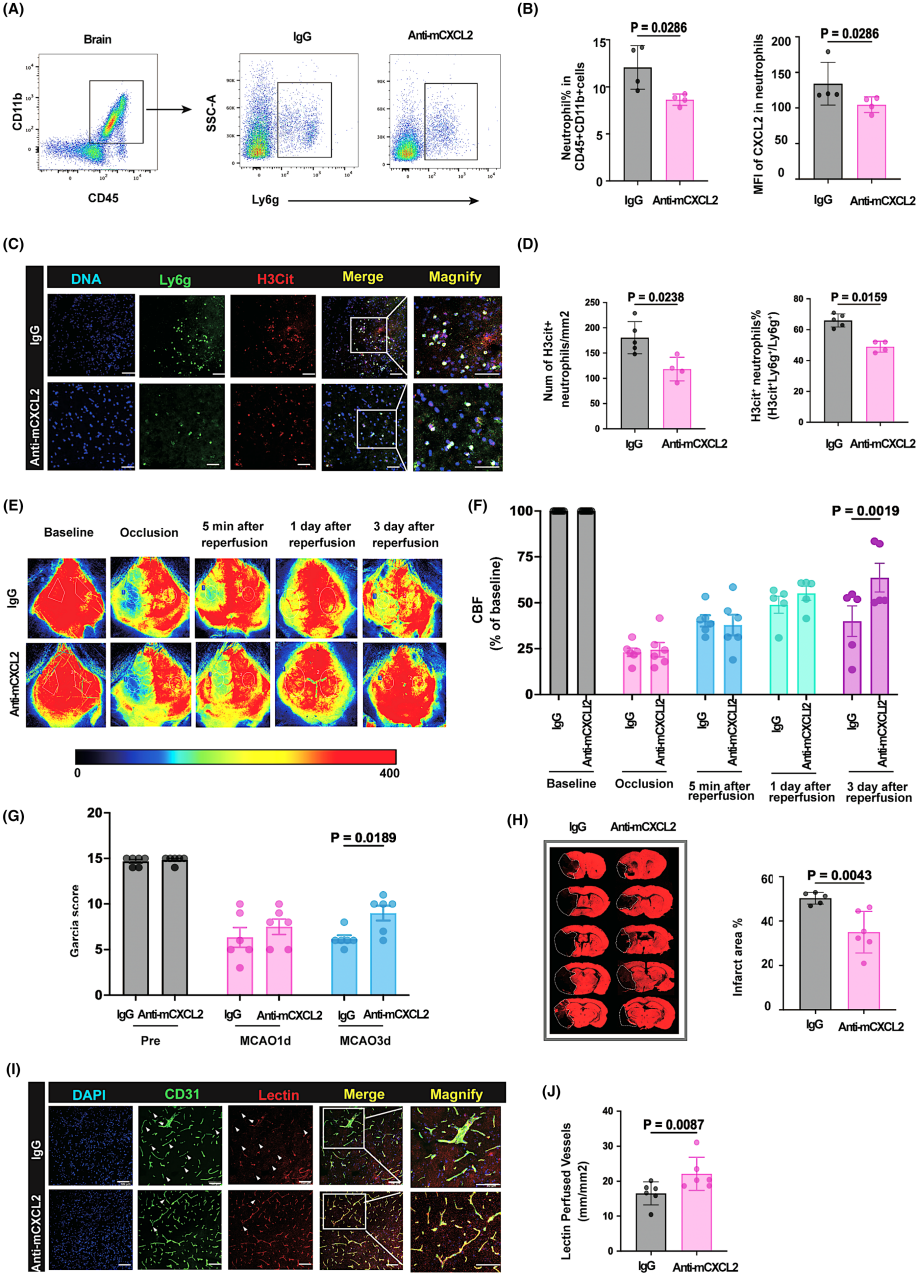
CXCL2 neutralization ameliorates ischemic brain injury and improves vascular reperfusion (A, B) flow cytometric analysis of infiltrated neutrophils in IgG‐ and anti‐mCXCL2‐treated MCAO mice, *n* = 4 mice per group; (C) representative images of coronal brain slices collected after tMCAO showing the DNA (DAPI, blue) localization of H3cit (red) in Ly6g^+^ (green) neutrophils in the core area. Scale bar: 20 μm; (D) quantification of the H3cit^+^Ly6g^+^ cells indicated in G; *n* = 4 mice per group. (E) representative images of cerebral blood flow (CBF) in IgG and CXCL2 neutralization antibody‐treated mice; (F) quantification of cerebral blood flow (CBF) in IgG and CXCL2 neutralization antibody‐treated mice, *n* = 5–6 mice per group; (G) Neurological deficit score (as indicated by Garcia score) was assessed during the first 3d after tMCAO, *n* = 5–6 mice per group; (H) infarct area at 3d after tMCAO was quantified on MAP2‐stained (red) coronal sections. Dashed lines define the infarct area, *n* = 5–6 mice per group; (I) representative images of CD31 (green) and lectin (red) immunostaining in the peri‐infarct cortex at 3 days after MCAO; (J) quantification of lectin (red) positive perfused vessels in the peri‐infarct cortex at 3 days after MCAO.

We next investigated whether CXCL2 neutralization could inhibit neutrophil infiltration and improve stroke outcome. Mice were injected with CXCL2‐neutralizing antibody (MAB452‐500, 25 μg/mouse) or its rat IgG2B isotype control (MAB0061‐500, 25 μg/mouse) 1 h after reperfusion. Flow cytometry analysis revealed that CXCL2 neutralization inhibited neutrophil infiltration into the ischemic brain and decreased CXCL2 expression in the infiltrated neutrophils (Figure 5A,B). Furthermore, we detected that administration of CXCL2‐neutralizing antibody reduced H3cit‐positive neutrophils compared with IgG control treatment in the core infarct region (Figure 5C,D). Cerebral blood flow and neurological function were assessed before measurement of brain infarction at 1 and 3 days after MCAO. Treatment with CXCL2‐neutralizing antibody improved blood reperfusion 3 days after MCAO (Figure 5E,F) and neurological deficit measured by Garcia score (Figure 5G) compared to IgG control treatment. Besides, CXCL2‐neutralizing antibody significantly reduced brain infarct (Figure 5H). Importantly, we further found that the perfused vessels labeled with tomato‐lectin and CD31 were also increased in the ischemic mouse brain treated with CXCL2‐neutralizing antibody compared to those treated with IgG2B control (Figure 5I,J). Together, these findings suggest CXCL2‐induced NETs formation by neutrophils, and that CXCL2 neutralization can enhance vascular reperfusion and improve stroke outcome.

## 4 DISCUSSION

In this study, we found that cerebral ischemic stroke triggered expansion of CXCL2^+^ neutrophils in the brain border, which were NETosed and potentially mediated incomplete reperfusion post‐ischemic stroke. Our results expand the understanding of the role of brain border‐associated myeloid cells in exacerbating ischemic stroke and propose novel treatment that targets CXCL2^+^ neutrophils for no‐flow‐related neurological deficits following cerebral ischemic stroke.

Reperfusion therapy is the first‐line treatment for acute ischemic stroke.^21^ However, clinical studies have shown that the proportion of patients with acute ischemic stroke who develop no‐reflow after reperfusion therapy ranges from 25% to 38%.^22^, ^23^ Therefore, the promotion of effective reperfusion and a favorable prognosis is a major concern for stroke patients. Multiple mechanisms may contribute to no‐reflow, including endothelial cell dysfunction, embolization of thrombus fragments into more distal vessels, pericyte death, and leukocyte adhesion.^21^ Elevated peripheral blood neutrophil counts are a biomarker of unfavorable prognosis for stroke patients treated with thrombolytic therapy.^24^, ^25^ This suggests that neutrophils may be involved in exacerbating tissue damage in stroke patients.^26^ However, how neutrophils are involved in incomplete reperfusion remains largely unknown.

Previous studies have shown that extracellular traps (NETs) released by neutrophils are positively correlated with poor prognosis in stroke.^27^ Marco Bacigaluppi et al. reported that the more severe no‐reflow phenomenon observed in aged mice was due to stroke‐induced increased emergency granulopoiesis and release of NETs within 48 h after stroke.^7^ Bing‐Qiao Zhao et al. observed that neutrophils isolated from ischemic mice formed more NETs after exposure to LPS and showed a stronger tendency to form NETs.^28^ The peak period of intravascular and parenchymal NET production by neutrophils was 3–5 days. The ablation of NETs with DNase 1 significantly reduced BBB injury and increased the formation of new functional vessels, suggesting that NET formation is a cause of vascular injury. In our study, we found that CXCL2 neutralization inhibited the formation of NETs by neutrophils, thereby improving vascular reperfusion 3 days after stroke.

The CXC motif chemokine ligand 2 (CXCL2) has been shown to be implicated in the pathogenesis of cardiovascular disease.^19^ As a potent chemokine that recruits neutrophils under inflammatory conditions, CXCL2 has been reported to be involved in the formation of atherosclerotic plaques.^29^ However, the exact mechanism by which CXCL2, as an important inflammatory chemokine, acts in cardiovascular disease remains unclear. In our study, we observed that CXCL2^+^ neutrophils of cerebral border origin had an enhanced ability to form NETs, which impaired vascular reperfusion after ischemic stroke. Lai guan Ng et al. found that neutrophils express high levels of CXCL2, which further amplifies neutrophil recruitment and activation in an autocrine and/or paracrine manner.^30^ In addition, CXCL2^+^ macrophages exhibit a high level of inflammatory chemotactic activity, and CXCL2^+^ macrophages exhibit a high level of inflammatory chemotactic activity.^31^ In addition, CXCL2^+^ macrophages exhibit a high level of inflammatory chemotactic activity. CXCL2^+^ macrophages exhibited a senescence‐associated secretory phenotype (SASP). SASP is a specific phenotype of senescent cells that express and secrete cytokines, chemokines, proteases, growth factors, and biologically active lipids^32^ and is a key driver of chronic inflammation and aging.^33^ We found that stroke induced an expansion of the CXCL2^+^ neutrophil phenotype in the meninges, and neutrophil maturation increased, suggesting that ischemic stroke‐induced CXCL2^+^ neutrophils at the brain border may also be an aging‐associated secretory phenotype. More studies are needed to understand the characteristics of CXCL2^+^ neutrophils.

In neuroinflammation, brain‐immune interactions fundamentally affect brain physiology.^34^ A deeper understanding of the brain's immune milieu is critical to the development of new therapies for treating neurological disorders. Recent studies have emphasized that brain borders are central to brain‐immune interactions.^35^ Immune cells reside at the borders of the central nervous system, including the choroid plexus, meninges, and perivascular spaces. These niches, as well as the meningeal lymphatic system and cranial microchannels, provide a direct pathway for interaction between the brain and the immune system.^36^ There is growing evidence that the cranium allows immune cells to rapidly enter the brain through microchannels.^37^ However, the mechanisms by which immune cells located at the brain's borders participate in the progression of the disease need to be further investigated. The meninges lie beneath the skull and are separated from it by an epidural cavity containing fat and blood vessels. The meningeal layer has been shown to contribute to local inflammation.^38^ There is evidence of an association between intact meningeal immunity, behavioral traits, and brain protection due to the fact that meningeal *γδ* T cells regulate anxiety by influencing glutamate‐releasing neurons in the cerebral cortex^39^ and that the aforementioned meningeal IgA^+^ plasma cells are critical for protecting the brain from fungal transmission. Recent studies have shown that the meninges provide a niche for the development of immature B cells that migrate from the cranial BM.^40^ Single‐cell RNA sequencing data by Zeynep Ilgin Kolabas et al.^4^ showed that the meninges produce a subpopulation of neutrophils after ischemic stroke that is absent from both the cranial and peripheral BM. This suggests that the meninges are not only a transit point for immune cells but also a gathering place for neutrophils, which rapidly differentiate into new cell subpopulations upon stimulation.

In summary, our results demonstrate that stroke‐induced brain border expansion and infiltration of CXCL2^+^ neutrophils which showed a great tendency to form NETs and dampened subsequent vascular reperfusion. Our study provides new insights that targeting CXCL2^+^ neutrophils generated by meninges could be a potential treatment for improving vascular reperfusion following ischemic stroke.

## FUNDING INFORMATION

P.L. is supported by the National Natural Science Foundation of China (NSFC, 91957111, 81971096, 82061130224, M‐0671, U22A20295), New Frontier Technology Joint Research sponsored by Shanghai Shenkang Hospital Development Center (SHDC12019102), Shanghai Municipal Education Commission‐Gaofeng Clinical Medical Grant Support (20181805), “Shuguang Program” supported by Shanghai Education Development Foundation and Shanghai Municipal Education Commission (20SG17), and “Shanghai Outstanding Academic Leaders Program” from Shanghai Municipal Science and Technology Committee (20XD1422400). P.L. is supported by a Newton Advanced Fellowship grant provided by the UK Academy of Medical Sciences (NAF\R11\1010). P.L. is also supported by the Innovative Research Team of HIGH‐level Local Universities in Shanghai (SHSMU‐ZLCX20211602).

## CONFLICT OF INTEREST STATEMENT

The authors declare that they have no competing interests.

## Supporting information


Figures S1–S3.

## Data Availability

The research data are available from the corresponding author on reasonable request.
