# Supplementary material for: Brain border‐derived CXCL2 + neutrophils drive NET formation and impair vascular reperfusion following ischemic stroke
Source: CNS Neurosci Ther. 2024 Aug 12;30(8):e14916. doi: 10.1111/cns.14916 (PMC11319398; doi:10.1111/cns.14916)
Supplement: Supplementary file 1 — Figures S1–S3. [file CNS-30-e14916-s001.docx]

**Supplementary Figures**

**FigureS1. Signature genes of cluster 5 expressed in the brain at day1 after MCAO**


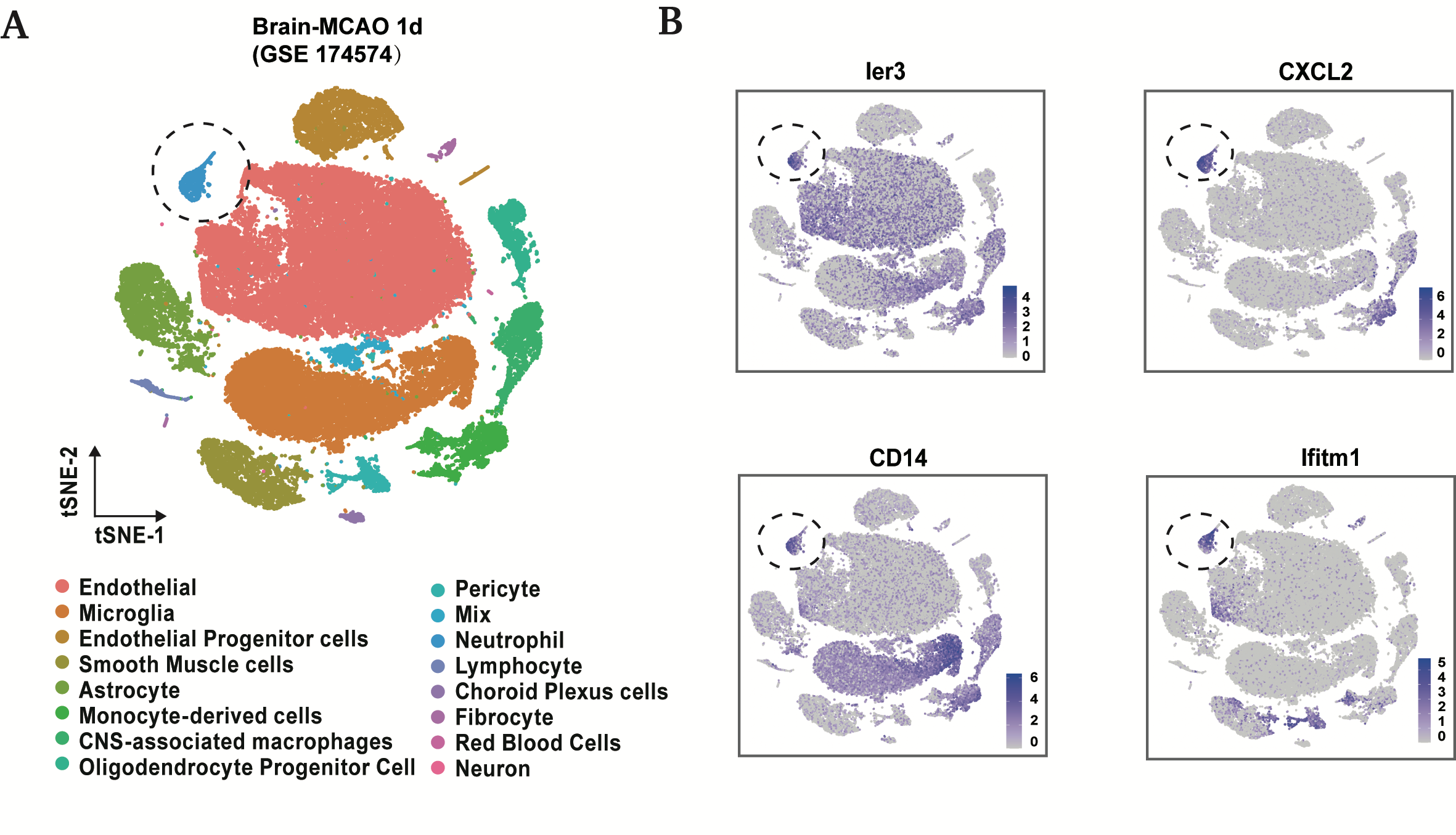


A. UMAP plot showing scRNA-seq transcriptomes of brain cells from sham and MCAO 1d mice (GSE174574); B. Expression of cluster 5 signature gene (Ier3, CXCL2, CD14 and Ifitm6) in clusters.

**FigureS2. Neutrophil localization**


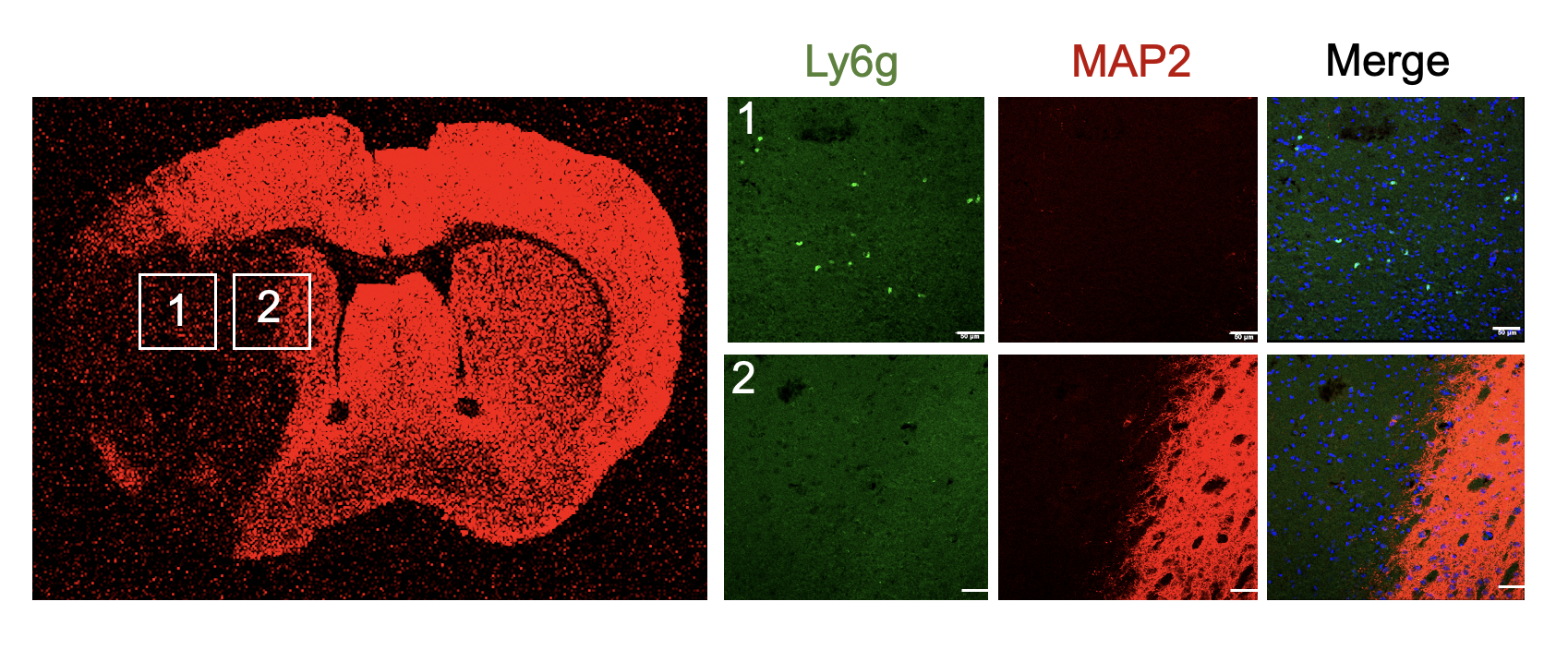


FigureS2. Infarct area was identified by staining for MAP2 (red). Neutrophils were labeled with Ly6g (green) in brain sections at day3 after MCAO.

**FigureS3. CXCL2 expressed in neutrophils from blood and calvaria**


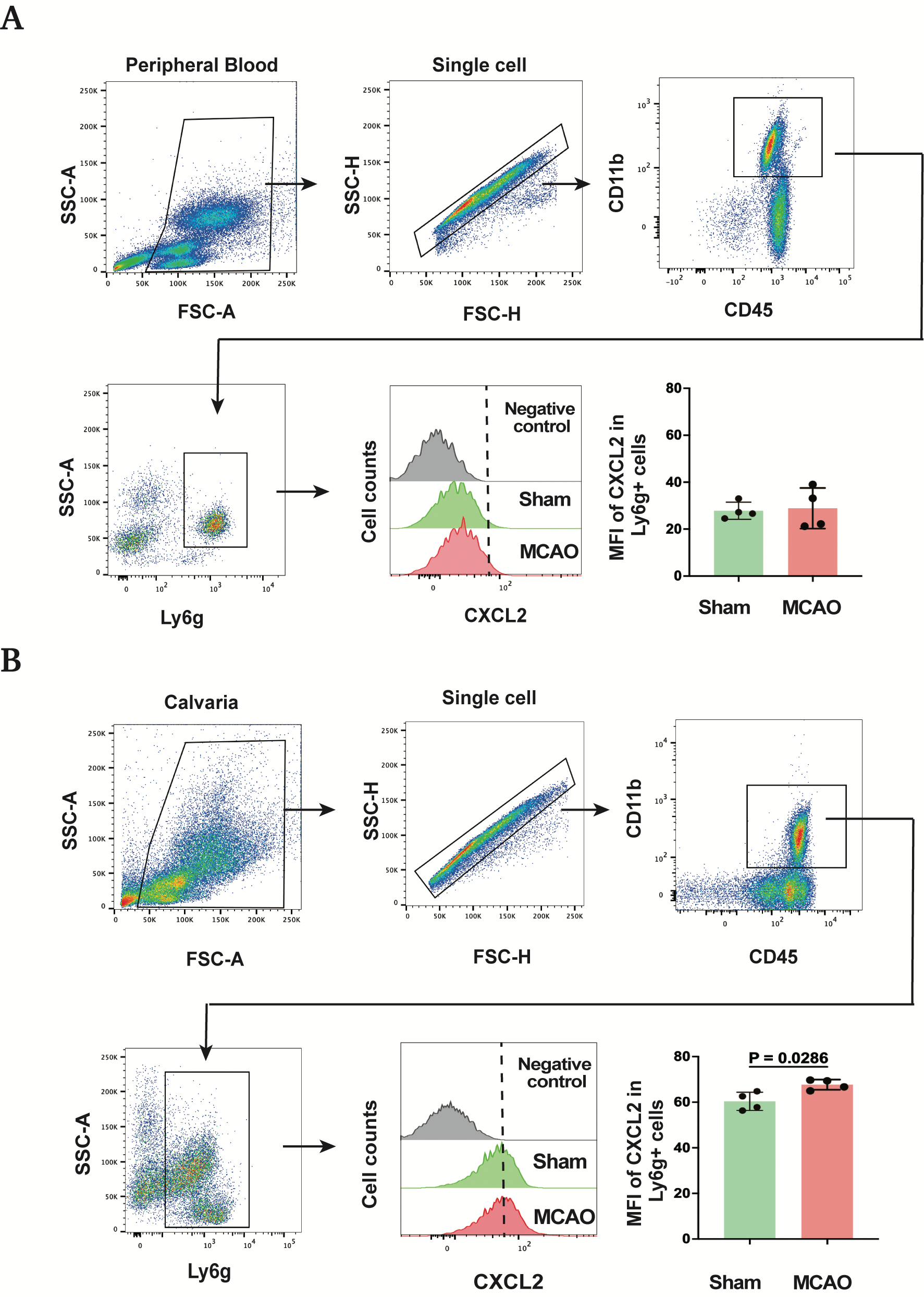


FigureS3. A. Flow cytometry analysis of CXCL2 expressed in neutrophils from peripheral blood in sham and MCAO 1d mice, n=4 per group; B. Flow cytometry analysis of CXCL2 expressed in neutrophils from calvaria in sham and MCAO 1d mice, n=4 per group.
